# Breaking barriers in HIV management: the rise of long-acting lenacapavir

**DOI:** 10.1097/MS9.0000000000003775

**Published:** 2025-09-02

**Authors:** Devya Khaim Chandani, Sahira Khan, Harshika Khaim Chandani, Muhammad Saad Khan, Abubakr Mahmoud

**Affiliations:** a Department of Medicine, Shaheed Mohtarma Benazir Bhutto Medical College (SMBBMC), Karachi, Pakistan; b Department of Medicine, Liaquat College of Medicine and Dentistry, Karachi, Pakistan; c Department of Medicine, Jinnah Sindh Medical University, Karachi, Pakistan; d Department of Medicine, University of Khartoum, Khartoum State, Sudan

**Keywords:** capsid inhibitor, HIV-1, lenacapavir, long-acting therapy, PrEP

## Abstract

Human immunodeficiency virus type 1 (HIV-1) remains a global health challenge, affecting over 38 million people as of 2024. The recent FDA approval of lenacapavir (Yeztugo®) on 18 June 2025 marks a significant advance in prevention and treatment. Lenacapavir is the first-in-class, long-acting HIV-1 capsid inhibitor, administered twice yearly via subcutaneous injection for pre-exposure prophylaxis in adults and adolescents ≥35 kg. It targets the capsid protein, disrupting multiple stages of the viral lifecycle without cross-resistance to existing antiretrovirals. Clinical trials (CAPELLA, CALIBRATE) demonstrated robust efficacy, with up to 94% viral suppression when combined with active agents and sustained CD4+ gains. Its prolonged half-life, minimal drug–drug interactions, and convenient dosing improve adherence, privacy, and healthcare access – critical in high-incidence, resource-limited settings. Ongoing PURPOSE trials are evaluating its role in high-risk populations. Lenacapavir’s favorable safety profile and public health potential support its integration into evolving HIV management and prevention strategies, potentially accelerating progress toward epidemic control.

*To the Editor,*

Human immunodeficiency virus type 1 (HIV-1) remains a major global health concern, characterized as a chronic viral infection that attacks the immune system, specifically CD4+ T cells, progressively compromising the body’s ability to fight off opportunistic infections. As of 2024, an estimated 38 million individuals are living with HIV worldwide, with more than 1.3 million new cases reported in the same year, underscoring the persistent and widespread nature of the epidemic^[1]^. Owing to limited treatment options for patients with multidrug-resistant HIV infection have led to the development of new therapy options^[2]^. This manuscript has been developed in accordance with the TITAN Guidelines (2025)^[3]^.

In this context, the recent FDA approval of Lenacapavir (Yeztugo®) on 18 June 2025, as a twice-yearly subcutaneous injection for pre-exposure prophylaxis, signifies a landmark in HIV prevention^[4]^. It is the first-in-class long-acting HIV-1 capsid inhibitor designed to reduce the risk of sexually acquired HIV-1 infection in adults and adolescents weighing at least 35 kg^[5]^.

It exhibits a unique multistage mechanism of action, targeting the HIV-1 capsid protein, a structural component critical for multiple stages of the viral lifecycle, i.e., inhibits capsid assembly and disassembly, blocks nuclear import of viral DNA, prevents reverse transcription and integration, and disrupts virion maturation without cross-resistance to existing antiretroviral classes. Its long half-life and minimal drug–drug interactions further enhance its clinical utility^[6]^.

The CAPELLA trial provides evidence in favor of treating HIV-1 with long-acting medications whose modes of action may reduce the emergence of resistance mutations^[7]^. The CALIBRATE trial shows 88% of patients who received lenacapavir experienced a reduction in viral load of ≥0.5 log_10_ over the 14-day functional monotherapy phase (compared to 17% on placebo; *P* < 0.0001), with a mean drop of – 1.93 log_10_ copies/mL, as opposed to – 0.29 log_10_ in the placebo group. HIV RNA levels were undetectable (less than 50 copies/mL) by Week 26 (81–83%), and they remained at ~83% by Week 52. The average CD4 cell increase among the participants was +81–83 cells/µL. Efficacy differed depending on the background regimen: 67% with no active drugs, 79% with one, and 94% with at least two active agents^[8]^.

From a public health perspective, the twice-yearly injection schedule not only improves convenience and privacy, important factors for individuals in stigmatized or resource-limited settings but also reduces the burden on healthcare systems by limiting clinic visits^[9]^. Current PURPOSE 1 and 2 trials are evaluating its effectiveness among high-risk populations, including adolescent girls and young women in sub-Saharan Africa, where HIV incidence remains disproportionately high^[10]^.

Safety data show lenacapavir to be well tolerated, with a few, mild side effects that can include injection site responses, headache, nausea, diarrhea, constipation, and stomach distention^[11]^.

In conclusion, lenacapavir offers a promising, long-acting alternative for both treatment-experienced patients and individuals seeking HIV prevention. Its unique pharmacologic profile, extended dosing interval, and favorable trial outcomes support its inclusion in evolving HIV management guidelines. As global efforts intensify to end the HIV epidemic, integrating lenacapavir into national and international prevention guidelines may play a pivotal role in achieving equitable and sustained protection for those at greatest risk. As research continues, lenacapavir may prove instrumental in achieving international goals for HIV eradication.

## Data Availability

No data were generated for this manuscript.
